# Towards a Visualizable, De-identified Synthetic Biomarker of Human Movement Disorders

**DOI:** 10.3233/JPD-223351

**Published:** 2022-10-14

**Authors:** Hao Hu, Dongsheng Xiao, Helge Rhodin, Timothy H. Murphy

**Affiliations:** a University of British Columbia, Department of Psychiatry, Kinsmen Laboratory of Neurological Research, Detwiller Pavilion, Vancouver, BC, Canada; b Djavad Mowafaghian Centre for Brain Health, University of British Columbia, Vancouver, BC, Canada; c Department of Computer Science, University of British Columbia, Vancouver, BC, Canada

**Keywords:** Artificial intelligence, computer-assisted diagnosis, computer-assisted image processing, neural networks (computer), movement disorders, Parkinson’s disease

## Abstract

Human motion analysis has been a common thread across modern and early medicine. While medicine evolves, analysis of movement disorders is mostly based on clinical presentation and trained observers making subjective assessments using clinical rating scales. Currently, the field of computer vision has seen exponential growth and successful medical applications. While this has been the case, neurology, for the most part, has not embraced digital movement analysis. There are many reasons for this including: the limited size of labeled datasets, accuracy and nontransparent nature of neural networks, and potential legal and ethical concerns. We hypothesize that a number of opportunities are made available by advancements in computer vision that will enable digitization of human form, movements, and will represent them synthetically in 3D. Representing human movements within synthetic body models will potentially pave the way towards objective standardized digital movement disorder diagnosis and building sharable open-source datasets from such processed videos. We provide a hypothesis of this emerging field and describe how clinicians and computer scientists can navigate this new space. Such digital movement capturing methods will be important for both machine learning-based diagnosis and computer vision-aided clinical assessment. It would also supplement face-to-face clinical visits and be used for longitudinal monitoring and remote diagnosis.

## INTRODUCTION

The field of computer vision has advanced considerably and approaches that recognize faces, handwriting, and digitize human form are now routine [1–4]. Yet, despite these tools becoming relatively commonplace, we are still reluctant to embrace them during the clinical assessment of movement disorders. There are many potential reasons for the slow adoption of computer vision and machine learning in movement disorders assessment. First, the size of training, validation, and testing datasets for movement disorders is limited. Due to privacy issues, individual patient data will require extensive data usage agreements before sharing within aggregate datasets [5]. Consequently, it requires either building an accurate diagnosis system from datasets with only a few examples or producing a representation of the patient’s data without privacy concerns. Second, there is concern about the robustness of clinical diagnosis systems. For example, medical machine learning systems can be misled by small perturbations like rotation or some invisible additive noise for humans [6]. A well-known example is that a panda will be recognized as a gibbon with high confidence after adding noise not perceptible to humans [7]. For Parkinson’s disease (PD), automated diagnosis based on detecting dysphonia dropped to 37% from 93% after similar in-perceptible perturbation [8]. Third, there are potential legal and ethical concerns about the black-box nature of machine learning and end-to-end solutions that bypass clinicians [9].

The objectives of our hypothesis are to present the emerging field of computer vision and provide cases for future adoption in clinical assessment of movement disorders. Using a synthetic patient interface where we represent human movements within a framework of 3D realistic human mesh-volumetric body models [10, 11], we hope that the size of available training, validation, and testing datasets can be increased (by sharing) and this will enable standardized digital movement disorder diagnosis. Importantly, capture of human 3D pose within an articulated 3D body model provides both path to visualization and quantification since body keypoints coordinates are available for further analysis and automated clinical severity scoring.

### Digital capture of human movements

The most instinctual usage of video recording is to capture movements. In clinical diagnosis, it would be as a permanent record of a motor assessment in response to the commands of a clinical expert. While there are methods to directly measure parkinsonian movements using accelerometers and other sensors that hold considerable promise [12, 13], the procedure of data capture is complex and requires specific devices with often limited bandwidth. The raw signals (such as angle, velocity, and acceleration) do not directly lead to a diagnosis of movement disorders and are hard to visualize and directly interpret by clinicians [14]. Therefore, approaches that align with current observer-based clinical motor assessment such as video recordings and motion capture are potentially more instinctive for clinicians than sensor-based methods. While such videos would be a necessary adjunct to the clinical record, standardization of parameters such as lighting, camera angle, field of view, and concern around privacy also may limit utility. Notwithstanding the drawbacks of video recording, advances in computer vision provide the means of capturing human form within a digital environment [15–17]. Here, previous research has established that biological motion and pose (disposition in space) can be represented using coordinates based on body keypoints (limbs, digits, etc.) [18]. There is also the possibility to represent these poses within the context of skeletal or volumetric body models [10, 19]. We suggest that human body synthetic models may provide a more generic solution enabling anonymization and standardization across centers necessary for future use of digitized movements as a clinical biomarker.

### What’s important for the diagnosis of movement disorders?

**Fig. 1:**
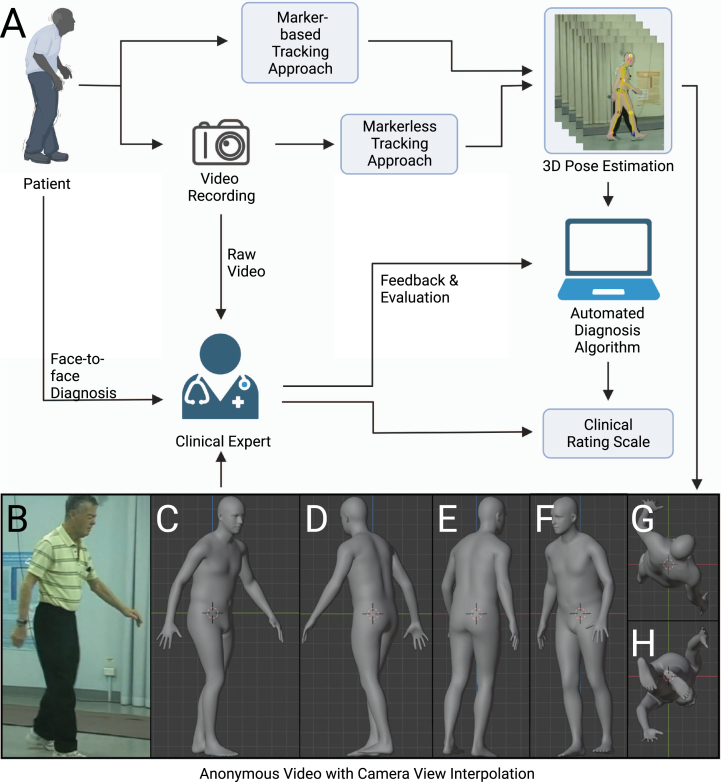
Scheme for clinical assessment with any movement-related clinical rating scale. The movement-related clinical rating scale has a pivotal role in the diagnosis of neurological diseases such as Parkinson’s disease. There are several possible ways to conduct an assessment of clinical rating scale: face-to-face assessment, remote assessment based on raw video recording, remote assessment based on anonymous videos, and automated assessment with algorithms. We argue that the anonymous video is of benefit to privacy protection, assessment, and algorithm design.

The diagnosis of movement disorders such as PD is rooted in patient and family history, symptom onset, and physical exam features [20]. Particular importance is placed on qualitative features of resting tremor, bradykinesia, rigidity, and gait disturbance as these symptoms can be followed to evaluate disease progression and treatment response using scoring by human observers. Typically, clinical movement disorder rating scales have clinical severity scores for both whole body posture and walking movements, but also more fine features such as movements of hands and fingers (tapping). To illustrate these points, we examine the Movement Disorder Society Unified Parkinson’s Disease Rating Scale (MDS-UPDRS) [21]. Computer vision and augmentation (interpolated 3D views) through the use of synthetic human form could be particularly useful for motor examination (part III) of the MDS-UPDRS (Fig. 1). Initial scales (MDS-UPDRS Sections 3.1-3.2) involve speech and facial expressions. Facial expressions are also potentially well suited to automatic analysis using higher resolution models [22, 23] or specific face models [3]. Some aspects of motor scales, such as Motor Examination in MDS-UPDRS Section 3.3, require assessment of rigidity using a clinical observer to manipulate limbs and the neck. Since they require observer action and patient feedback, it is unlikely that these components of motor scales could be fully automated. Computer vision could potentially contribute to most of the other aspects: on the finest level, cameras optimized to report finger tapping would be necessary for Motor Examination in MDS-UPDRS Section 3.4 and lower resolution full body capture for Motor Examination in MDS-UPDRS Sections 3.5 and 3.6 that involve aspects of locomotion and transitioning from seated to standing posture. Re-creation of complete clinical rating scales from a single camera view is not possible as it will require subjects to perform tasks not suited to computer vision-based analysis or will involve different levels of resolution. Conceivably, a subset of movement-related phenomena that are well suited to computer vision could be used to predict the overall MDS-UPDRS score [24] or treatment response [25] providing some form of clinical guidance but still requiring interactive tests in other domains.

### Video monitoring of Parkinson’s disease to refine clinical diagnosis

There are many challenges in idiopathic parkinsonism, such as differential diagnosis from Parkinson-plus syndromes [26, 27]. While most physicians may readily notice tremor, bradykinesia, and postural instability, there is currently no test that can effectively confirm the diagnosis of idiopathic PD. At initial assessment, the main focus is put on confirming cardinal signs in PD [20]. However, the clinical course of the illness may reveal it is not PD, requiring that the clinical presentation be periodically reviewed to confirm the accuracy of the diagnosis [20, 28]. When PD diagnoses are checked by autopsy, on average, movement disorders experts are found to be 80% accurate at initial assessment and 84% accurate after they have refined their diagnoses at follow-up examinations [29]. Capture of human 3D pose within an articulated 3D body model can facilitate the remote diagnosis and longitudinal monitoring of motor function to promote diagnostic accuracy.

### Towards the diagnosis of movement disorders based on video recording

A typical diagnosis procedure of movement disorders based on video recording could involve the following steps: capturing human motion from videos, analyzing motion trajectories with diagnosis algorithms or experts, and evaluating the result in light of the patient’s clinical history and presentation (Fig. 1). Here we review the steps that form the basis for diagnosis within any movement-related clinical rating scale. We also introduce the use of synthetic human form that enables the expert to be remote or diagnose at a later time, as the anonymized data can be stored and sent without risks to privacy [30].

**Fig. 2:**
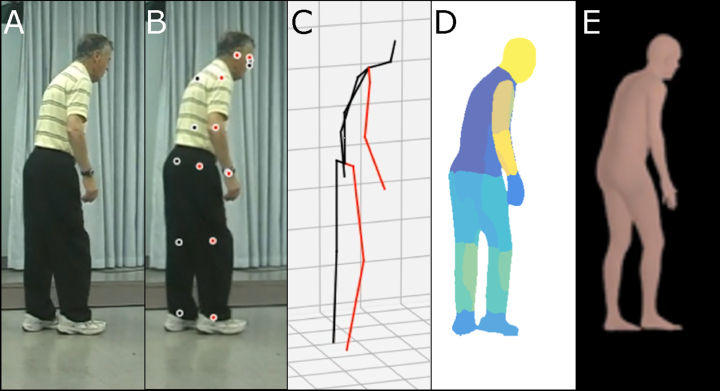
Illustration of synthetic human body modeling methods. A) Raw video image from open-source examples of Parkinson’s disease characterization through postural assessment. This video was obtained with permission from publisher Wiley [57]. B) Keypoints overlaid on video images. C) Skeletal representation. D) Body-parts-based representation. E) 3D volumetric representation.

A variety of methods have been proposed to capture human pose and motion necessary for diagnosis of movement disorders. In this hypothesis, the term ‘human pose estimator’ will be used in its broadest sense to refer to all computer vision-based algorithms to capture human pose and motion. A human pose estimator can be categorized into marker-based and markerless approaches. A marker-based approach requires subjects to wear fiducial markers on a specialized bodysuit or attached to their clothes [31, 32]. Disadvantages of this approach include demands for markers themselves, additional complexity, as well as concerns around sterilization and use across subjects. The markerless approach involves using a pre-trained deep neural network (see Supplementary Material for a glossary) to automatically identify joints and other keypoints of subjects [33, 34]. Although it can be slightly less accurate than the marker-based approach, advantages of markerless approaches include generalizability to different clothing and background and ease of use. A markerless approach can be classified based on how it virtually represents the human body: including a skeletal model with keypoints [2], a 2D human body model [35], or a 3D human body model (volumetric model) [1, 36, 37] (Fig. 2). Human synthetic body models are potentially adaptable to any population including elderly patients with movement disorders. One of the most widely used models, SMPL, was learned from the CAESAR dataset. The CAESAR dataset consists of a total of 3800 3D body scans collected from American and European civilians whose age ranges from 18 to 65 [38]. The latest upgraded version of the SMPL model, STAR, is able to account for differences in body mass index by learning from both CAESAR and SizeUSA datasets [11]. The SizeUSA dataset was collected from 2845 male and 6434 females with ages varying between 18 to 66 + providing diversity that may better match clinical populations [39]. The output of a human pose estimator is movement data (a series of vectors) that can be overlaid on the original raw video (see Supplementary Video 1). The movement data can then be analyzed to examine aspects of motor behaviors. Reducing video data to a series of vectors greatly simplifies the complexity and the computational cost of analysis.

We have focused on models necessary for automated diagnosis. It is also necessary to emphasize the importance of training, validation, and testing dataset scale and quality. Potentially, the “no free lunch” theorem holds for any algorithm [40], where any improvement in performance on one task must be paid for by lesser performance on other tasks. In contrast, the scale of datasets is a universal driving factor of performance, and it has some unique features [41]. First, the scale of data is often more important than the quality of the data. Second, there is a positive logarithmic relationship between the performance of computer vision algorithms and the scale of datasets. In summary, the scale of the training dataset guarantees the lower bound of the algorithm’s performance. On the other hand, garbage in, garbage out: the quality of the dataset limits the upper bound of the algorithm’s performance. A positive correlation was found between the resolution of video recording and performance of pose estimation for normal human subjects [42]. Motion blur and occlusion can also result in false pose detection and should be avoided or compensated for with both normal and PD datasets [43]. In general, the quality of any training, validation, and testing dataset can be assessed from the following dimensions: accuracy, completeness, redundancy, reliability, consistency, usefulness, and confidentiality [44]. In the context of PD datasets, these criteria could refer to the accuracy of 3D pose, completeness of patient information, reliability of MDS-UPDRS score, consistency in data format, etc.

In human pose estimation, large-scale training, validation, and testing datasets use marker-based tracking and triangulation from multiple cameras to place subjects within a 3D coordinate system and are typically captured in a specialized studio. The most widely used datasets for 3D human pose estimation model training, validation, and testing are: HumanEva, Human3.6M, MPI-INF-3DHP, TotalCapture, 3DPW, and AMASS [31, 45–50]. These datasets include images, videos, and annotations. Annotations can include: 2D poses, positions of keypoints in 3D space, types of activities, and 3D body scans corresponding to images (Table 1). To date, one of the major obstacles of applying any computer vision approach to movement disorders related data is access to training, validation and testing datasets that reflect both the scale and diversity of expected clinical populations.

**Table 1:** Summary of datasets on 3D human pose estimation

Datasets	HumanEva [46]	Human3.6M [45]	MPI-INF-3DHP [47]	TotalCapture [31]	3DPW [50]	AMASS [48]
Number of subjects	4	11	8	5	7	344
Number of poses	37000	3.6 M	1.3 M	1892176	51000	11265
Types of actions	6	15	8	4	–	–
Sex ratio	–	6 : 5	4 : 4	4 : 1	–	–
Age	–	–	–	–	–	–
BMI	–	17–29	–	–	–	–

### Comparing the performance of human pose estimators

Neural network pose estimators are trained, validated, and tested on a large example set collected in a controlled studio. To be generalizable, they need to be adapted to apply to human motion data, collected from any scene, performed by different objects. Domain adaptation can broadly be defined as the ability of a model trained on one dataset to still perform well when employed on different but relevant datasets. Existing research has recognized the critical role of domain adaptation and investigated the performance of 3D human pose estimators in the context of wild settings (non-studio or controlled environment) [51, 52], but in the absence of motor impairments.

In most recent studies, deep neural networks have dominated the field of human pose estimation [34]. Those algorithms can be coarsely classified according to their input and output. The explicit structure of deep neural networks can be designed to work with different inputs and outputs. Generally, the input of a deep neural network can be one single frame (frame based) or a sequence of successive frames (video based). The output can be keypoints that depict joints of the human body (skeletal), parameters that control an existing 3D model of the human body (volumetric model based) or pointcloud which directly represent the human body in 3D space (pre-built modelfree).

To compare the different algorithms that could be applied to video of PD patients, we choose densepose3D (pre-built model free), VIBE (video based), and PyMAF (frame based) as these represent the most accurate tools for volumetric based methods of human pose estimation to date [1, 23, 37]. As a comparison, we report accuracy with VideoPose3D, a well-used skeletal method (keypoints model) that can serve as a benchmark measurement [2]. We list the performance and features of different 3D human pose estimators [1, 2, 23, 37] on large scale human 3D pose datasets of healthy people [45] in Table 2. The performance was measured using mean per joint position error (MPJPE) in mm on Human3.6M datasets (see Supplementary Material for a glossary). Human3.6M datasets consist of 3.6 million different human poses taken during 15 daily activities like walking, sitting, eating, etc. [45]. The poses were collected with a motion capture system and its corresponding image recorded from 4 camera views. The motions in Human3.6M datasets were performed by 11 healthy professional actors, 5 female and 6 male, whose BMI ranges from 17 to 29. Poses from 2 male and 2 female motions were kept as testing datasets. A limitation is that demographic information like age and ethnicity was not reported. It is significant that most reported error measurements are relatively small compared to expected changes during movements such as walking or stooped posture that characterize motor impairments, with the exception of tremor and bradykinesia (see below). Nonetheless, it is conceivable that error values reported for commonly used algorithms may not necessarily be generalizable to all subjects, in particular those affected by movement disorders such as PD. Therefore, we also evaluated the performance of different pose estimators [1, 2, 36, 53–56] on available open-source movement disorders videos [57] collected under “wild settings” that could represent video captured without specialized equipment within a clinical environment (Table 3, see Supplementary Video 1). The open-source movement disorders videos from a male subject late 70 s with advanced PD with sporadic responses to oral levodopa. The videos were taken in the “off” state. In the absence of these motion-capture studios and 3D ground truth (see Supplementary Material for a glossary), we projected the information from 3D back to 2D and examined its alignment relative to 2D images. We adapted two metrics in 2D human pose estimation to assess the reprojection accuracy, the percentage of correct keypoints (PCK) and the area under the curve (AUC) [58, 59]. The accuracy of different human 3D pose estimators (assessed using reprojection) was up to 90% when compared to the human ground truth or a computer vision-based algorithm DeepLabCut (DLC) [53] and, as expected, was lower for all methods when expressed as the more stringent AUC (Table 3). It will be important to estimate how well the volumetric models trained on large groups of normal individuals transfer to PD patients; so far, our results with 2D video to 3D volumetric models (Table 3) indicate that this will work. These results must be interpreted with caution because the pose data is likely limited to only gait and gross body posture analysis. It has previously been observed that rating tremor and bradykinesia with video recording can be difficult for even well-trained human raters [60, 61]. In contrast, gait and postural stability analysis based on video recording has strong agreement between human raters [60, 61]. This difficulty may be due to technology limitations in video recording using webcam or cell phone cameras: such as camera motion blur, single view angle, resolution, etc. However, a full discussion of camera related issues is beyond the scope of this study.

**Table 2:** Performance of 3D human pose estimators for normal human subjects based on published values

	Skeletal model	Volumetric model		
Algorithms	VideoPose 3D [2]	Densepose3D [37]	VIBE [1]	PyMAF [23]
Error on Human3.6M (MPJPE)	46.8 mm	113.6 mm	65.9 mm	57.7 mm
Features	Simple, fast, less bias	Do not require pre-built 3D model	Video-based, Successful implement in movement disorder diagnosis	Frame-based, Supports single image
Disadvantages	Unable to capture small repetitive body movements, lack of shape information	Lower accuracy, High computational cost	Over-parameterization, likely overfitting (see Supplementary Material for a glossary)	
Links for code	https://github.com/facebookresearch/VideoPose3D	–	https://github.com/mkocabas/VIBE	https://hongwenzhang.github.io/pymaf

**Table 3:** Calculated reprojection accuracy of different human pose estimators

Algorithms		Deep LabCut	Open pose	Detectron2 + Mask RCNN	Video Pose3D	VIBE	PyMAF
Using human labeling as the ground truth 100% (1 video clip, see Supplementary Video 1)	Mean PCK@0.2	97.71%	90.00%	92.04%	79.62%	92.33%	95.24%
	Mean AUC on PCK@0.2	74.51%	55.85%	56.34%	38.08%	47.85%	52.86%
Using DeepLabCut as the ground truth 100% (across 10 video clips)	Mean PCK@0.2	–	–	87.32%	82.11%	85.25%	89.57%
	STD PCK@0.2	–	–	5.15%	7.18%	6.81%	6.64%
	Mean AUC on PCK@0.2	–	–	53.09%	39.76%	42.85%	49.31%
Links for Codes		https://github.com/DeepLabCut/DeepLabCut	https://github.com/CMU-Perceptual-Computing-Lab/openpose	https://github.com/facebookresearch/detectron2	https://github.com/facebookresearch/VideoPose3D	https://github.com/mkocabas/VIBE	https://hongwenzhang.github.io/pymaf/

### Challenges and opportunities with digital movement capture and analysis.

There are also multiple challenges associated with video analysis. Although all three model types (skeletal models, 2D human body models, and volumetric models) (Fig. 2C-E) satisfy ethical concerns around patient anonymity, only volumetric models are particularly suited for qualitative clinical diagnosis by expert observers because they have the appearance of human subjects. However, volumetric models could suffer from inherent biases and assumptions about shape, which are built into the model. In terms of computational load, the skeletal model requires less processing and would be the most efficient, but lacks human appearance. Overall, by having data within the context of 3D models, observers have additional flexibility with respect to viewing angle for potentially more accurate scoring, or the ability to match view angles from different studies or sites. It is noteworthy that artifacts could be introduced due to camera occlusion and other factors. Furthermore, tremors and small repetitive body movements associated with PD would be within the error of most human pose estimators using the most advanced commercial video capture systems [25]. Alternatively, depth-sensing motion ability (like Azure Kinect, Intel RealSense, etc.) [62, 63] or motion sensing devices such as accelerometers [12, 13] might be better suited for this use case and used as an adjuvant to video scoring. As human movement recording equipment and pose estimation algorithms advance, we anticipate these techniques can be used to collect visualizable, de-identified biomarkers of human movement disorders. First, digitally captured movement disorder data allows anonymization of datasets which will be a vehicle for data sharing and future collaboration by placing the data within a common synthetic format. It can relieve the problem of insufficient data during training of neural networks. Second, it provides a vehicle for re-evaluation by multiple clinical raters. Previous studies have shown interrater variability within the MDS-UPDRS: human raters often disagree on the exact severity of a single patient [24, 64]. With digitally captured data and the use of volumetric models, it will be possible to potentially elicit second opinions on ambiguous findings from re-analysis by human raters. Third, collection of raw video data and then conversion to a synthetic format may help remove bias clinicians could have if they were trained on a patient population which was not necessarily representative of all current patients. For example, synthetic models can help remove the influence of factors due to the clothing or lighting associated with particular patient image acquisition. Conversion of movements to a standard body plan may also reduce ambiguities around a person of extreme variation within body mass index (BMI), although synthetic models also exist that can represent BMI. Fourth, video capture and the use of robust synthetic methods for anonymization could potentially provide the opportunity to capture variation in movement chronically within the patient homes. These assessments combined with robust algorithms to differentiate a normal from disease-based movements could be useful in detection of early pre-manifest events which would be otherwise rare to observe within a short typical clinical assessment.

There are more challenges in the later stages of PD, as most PD patients eventually need levodopa and later develop levodopa-induced fluctuations and dyskinesias [28, 65]. In this case, the aim is to reduce PD symptoms while controlling fluctuations in the effect of the medication [28]. Instead of periodically following up in the clinic, a better solution may be longitudinal monitoring of motor fluctuations and dyskinesias in patient-own environments. However, some limitations are expected in patient-own environments during longitudinal monitoring. First, the major cause of occlusion will be furniture instead of clinicians and other patients. Second, more variability in illumination is caused by daylight instead of institutional lighting. Third, the recording devices in patient-own environments are consumer-grade devices like webcams, cellphones, and surveillance cameras. The resolution and sampling rate may result in inability to capture high frequency movement like tremors. Similarly, long shutter speeds can lead to motion blur. To relieve those problems to a certain degree, multiple cameras can be employed for better results. We suggest that patients and providers could fix with tripods and standardized pipelines for video capture aided by known fiducial objects. In the absence of calibration, advanced machine learning approaches could approximate spatial calibration and perspective from known structures such as walls and the orientation of the patient within the assumed vertical axis.

### Going from movement data to diagnosis and treatment

Once human poses and movements (pose sequences) are obtained, algorithms can further align findings to clinical rating scales and alert the clinician to pay attention to the abnormal movements presented in the video. To date, several studies have had some success for aligning movement data with clinical rating scales [66–68]. To address rater variability, one can employ focal neural networks and introduce a regularization (see Supplementary Material for a glossary) term such as a rater confusion estimation which encodes the rating habits and patterns of different raters [24]. Using this approach, an automated video-based PD diagnosis algorithm reached an accuracy of 72% for consistency with the majority rater vote and an accuracy of 84% for consistency with at least one of all raters when predicting MDS-UPDRS scores for gait and finger tapping. Although recent work suggests markerless video approaches have had some success for PD [24, 25, 68, 69], overall accuracy could be improved when compared to sensor-based approaches [12, 70]. We should emphasize that there are minimal potential barriers for application as most models are pre-trained requiring little user input.

Accurate diagnosis of PD requires assessment of both dynamic properties of human pose such as gait analysis, but also static properties like postural deformities and dystonia. For example, in the early stages of PD, rigidity is often asymmetrical and tends to affect the neck and shoulder muscles prior to the muscles of the extremities [71]. The continuous contraction of muscles may cause typical posture of the body [20, 72]. We assert that static postural changes would be ideally quantified using automated methods that could potentially be less subject to limitations around bandwidth associated with motion capture. Previous work in pose estimation was able to identify differences within subjects based on individual static images [73, 74]. The patient may also manifest mask-like facial expression (hypomimia, a reduced degree of facial expression) [20]. The facial expression is not easy to track by keypoints-based methods (that mark discrete body parts), but recent expressive body recovery methods, such as PIXIE [75], show promising results that can reconstruct an expressive body with detailed face shape and hand articulation from a single image.

Current automated systems assist diagnosis instead of bypassing clinicians and efforts to improve their interpretability have been minimal. Intuition, pattern recognition, and clinical training are an important part of any movement disorders diagnosis. We emphasize that the use of synthetic data does not exclude the use of supervised machine learning approaches that have been trained based on labels derived from the intuition of clinical experts. As digital movement capture methods become more routine, more data will be gathered within common formats. By combining these evolving datasets with existing everyday human activities datasets like MPII and Human3.6M [45, 76], it makes measuring the deviation from normal movements plausible. It is conceivable that this could be used as a screening tool to assist clinician diagnosis by highlighting suspicious movements, and examining at-risk individuals leading to earlier symptom identification, and potential lifestyle or treatment options being implemented proactively. It also benefits the development of a human interpretable algorithm in quantitative diagnosis. Although machine learning models have been successfully applied in medical imaging, these methods often fail to convince users because of a lack of transparency, interpretability, and visualization. Human interpretability (see Supplementary Material for a glossary) is fundamental to developing a reliable and understandable diagnosis algorithm for clinical use since the user might be misled by incorrect prediction of a black box system [77, 78]. Interpretability can be enhanced when the algorithm can weight model features based on known human body parts [79]. With a transparent and interpretable algorithm, users can decide whether the prediction for a body part of interest is reasonable or not. However, human interpretability is hard to quantify. We suggest the volumetric representation could be used as a visualization tool during the collection of human feedback, such as anonymized evaluation by expert raters.

As the disease advances, deep brain stimulation (DBS) has been used to reduce motor symptoms in severe cases where drugs are ineffective and cause side effects [28, 80]. In this case, the video-based 3D body model approach can be used to create body maps of motor changes for a patient and can serve as a visualization tool for physicians and open the opportunity for remote modulation of DBS parameters.

### Conclusion and future directions

We anticipate that the use of synthetic human form may aid the uptake of movement data as an open-source biomarker. Synthetic human form also provides an ideal vehicle for the generation of augmented training data for use within next-generation computer vision algorithms [81–83]. Animal studies have already taken advantage of a synthetic mouse model [84] to generate training data for pose estimation. In other work, a synthetic full body drosophila model [85] permits virtual force measurements. We anticipate that human 3D body models will be both tools for addressing relevant physiological questions concerning human form and providing specific forms of training, validation and testing datasets that lead to improved quantification of both normal function and disease.

## Supplementary Material

Supplementary Material

Supplementary Video Click here for additional data file. Illustration of different pose estimation methods.

## References

[1] Kocabas M , Athanasiou N , Black MJ (2020) VIBE: Video inference for human body pose and shape estimation., 2020 IEEE/CVF Conference on Computer Vision and Pattern Recognition (CVPR).

[2] Pavllo D , Feichtenhofer C , Grangier D , Auli M (2019) 3D human pose estimation in video with temporal convolutions and semi-supervised training., 2019 IEEE/CVF Conference on Computer Vision and Pattern Recognition(CVPR).

[3] Li T , Bolkart T , Black MJ , Li H , Romero J (2017) Learning a model of facial shape and expression from 4D scans. ACM Trans Graph 36, 1–17.

[4] De Campos TE , Babu BR , Varma M , Others (2009) Character recognition in natural images. VISAPP (2) 7, 2.

[5] Dove ES , Phillips M (2015) Privacy law, data sharing policies, and medical data: a comparative perspective. In, Medical Data Privacy Handbook, Gkoulalas-Divanis A, Loukides G, eds. Springer International Publishing, Cham, pp. 639–678.

[6] Finlayson SG , Bowers JD , Ito J , Zittrain JL , Beam AL , Kohane IS (2019) Adversarial attacks on medical machine learning. Science 363, 1287–1289. 30898923 10.1126/science.aaw4399 PMC7657648

[7] Goodfellow IJ , Shlens J , Szegedy C (2014) Explaining and harnessing adversarial examples. arXiv [statML]. 10.48550/arXiv.1412.6572

[8] Huai M , Zheng T , Miao C , Yao L , Zhang A (2022) On the robustness of metric learning: an adversarial perspective. ACM Trans Knowl Discov Data 16, 1–25.

[9] Razzak MI , Naz S , Zaib A (2018) Deep learning for medical image processing: overview, challenges and the future. In Classification in BioApps: Automation of Decision Making Dey N , Ashour AS , Borra S , eds. Springer International Publishing, Cham, pp. 323–350.

[10] Loper M , Mahmood N , Romero J , Pons-Moll G , Black MJ (2015) SMPL: a skinned multi-person linear model. ACM Trans Graph 34, 1–16.

[11] Osman AAA , Bolkart T , Black MJ (2020) STAR: Sparse Trained Articulated Human Body Regressor. In Computer Vision–ECCV 2020 , Springer International Publishing, pp. 598–613.

[12] San-Segundo R , Zhang A , Cebulla A , Panev S , Tabor G , Stebbins K , Massa RE , Whitford A , de la Torre F , Hodgins J (2020) Parkinson’s disease tremor detection in the wild using wearable accelerometers. Sensors 20, 5817. 33066691 10.3390/s20205817 PMC7602495

[13] Rigas G , Tzallas AT , Tsipouras MG , Bougia P , Tripoliti EE , Baga D , Fotiadis DI , Tsouli SG , Konitsiotis S (2012) Assessment of tremor activity in the Parkinson’s disease using a set of wearable sensors. IEEE Trans Inf Technol Biomed 16, 478–487. 22231198 10.1109/TITB.2011.2182616

[14] Kubota KJ , Chen JA , Little MA (2016) Machine learning for large-scale wearable sensor data in Parkinson’s disease: Concepts, promises, pitfalls, and futures. Mov Disord 31, 1314–1326. 27501026 10.1002/mds.26693

[15] Jovanov E , Hanish N , Courson V , Stidham J , Stinson H , Webb C , Denny K (2009) Avatar — A multi-sensory system for real time body position monitoring. 2009 Annual International Conference of the IEEE Engineering in Medicine and Biology Society. 10.1109/IEMBS.2009.5334774 19964961

[16] Zampogna A , Manoni A , Asci F , Liguori C , Irrera F , Suppa A (2020) Shedding light on nocturnal movements in Parkinson’s disease: evidence from wearable technologies. Sensors 20, 5171. 32927816 10.3390/s20185171 PMC7571235

[17] Dong Z , Guo C , Song J , Chen X . PINA: Learning a personalized implicit neural avatar from a single RGB-D video sequence. Proc Estonian Acad Sci Biol Ecol.

[18] Johansson G (1973) Visual perception of biological motion and a model for its analysis. Percept Psychophys 14, 201–211.

[19] Han F , Reily B , Hoff W , Zhang H (2017) Space-time representation of people based on 3D skeletal data: A review. Comput Vis Image Underst 158, 85–105.

[20] Jankovic J (2008) Parkinson’s disease: clinical features and diagnosis. J Neurol Neurosurg Psychiatry 79, 368–376. 18344392 10.1136/jnnp.2007.131045

[21] Goetz CG , Tilley BC , Shaftman SR , Stebbins GT , Fahn S , Martinez-Martin P , Poewe W , Sampaio C , Stern MB , Dodel R , Dubois B , Holloway R , Jankovic J , Kulisevsky J , Lang AE , Lees A , Leurgans S , LeWitt PA , Nyenhuis D , Warren Olanow C , Rascol O , Schrag A , Teresi JA , van Hilten JJ , LaPelle N (2008) Movement Disorder Society-sponsored revision of the Unified Parkinson’s Disease Rating Scale (MDS-UPDRS): Scale presentation and clinimetric testing results. Mov Disord 23, 2129–2170. 19025984 10.1002/mds.22340

[22] Pavlakos G , Choutas V , Ghorbani N , Bolkart T , Osman AA , Tzionas D , Black MJ (2019) Expressive body capture: 3D hands, face, and body from a single image. 2019 IEEE/CVF Conference on Computer Vision and Pattern Recognition (CVPR).

[23] Zhang H , Tian Y , Zhou X , Ouyang W , Liu Y , Wang L , Sun Z (2021) PyMAF: 3D human pose and shape regression with pyramidal mesh alignment feedback loop. 2021 IEEE/CVF International Conference on Computer Vision(ICCV).

[24] Lu M , Zhao Q , Poston KL , Sullivan EV , Pfefferbaum A , Shahid M , Katz M , Kouhsari LM , Schulman K , Milstein A , Niebles JC , Henderson VW , Fei-Fei L , Pohl KM , Adeli E (2021) Quantifying Parkinson’s disease motor severity under uncertainty using MDS-UPDRS videos. Med Image Anal 73, 102179. 34340101 10.1016/j.media.2021.102179 PMC8453121

[25] Martinez HR , Garcia-Sarreon A , Camara-Lemarroy C , Salazar F , Guerrero-Gonzáalez ML (2018) Accuracy of markerless 3D motion capture evaluation to differentiate between on/off status in Parkinson’s disease after deep brain stimulation. Parkinsons Dis 2018, 5830364. 30363689 10.1155/2018/5830364 PMC6180930

[26] Poewe W , Wenning G (2002) The differential diagnosis of Parkinson’s disease. Eur J Neurol 9 Suppl 3, 23–30. 10.1046/j.1468-1331.9.s3.3.x 12464118

[27] Connolly BS , Lang AE (2014) Pharmacological treatment of Parkinson disease: a review. JAMA 311, 1670–1683. 24756517 10.1001/jama.2014.3654

[28] National Collaborating Centre for Chronic Conditions (Great Britain) (2006) Parkinson’s disease: national clinical guideline for diagnosis and management in primary and secondary care, Royal College of Physicians. 21089238

[29] Rizzo G , Copetti M , Arcuti S , Martino D , Fontana A , Logroscino G (2016) Accuracy of clinical diagnosis of Parkinson disease: A systematic review and meta-analysis. Neurology 86, 566–576. 26764028 10.1212/WNL.0000000000002350

[30] Omberg L , Neto EC , Perumal TM , Pratap A , Tediarjo A , Adams J , Bloem BR , Bot BM , Elson M , Goldman SM , Kellen MR , Kieburtz K , Klein A , Little MA , Schneider R , Suver C , Tarolli C , Tanner CM , Trister AD , Wilbanks J , Ray Dorsey E , Mangravite LM (2022) Remote smartphone monitoring of Parkinson’s disease and individual response to therapy. Nat Biotechnol 40, 480–487. 34373643 10.1038/s41587-021-00974-9 PMC12812035

[31] Trumble M , Gilbert A , Malleson C , Hilton A , Collomosse J (2017) Total capture: 3D human pose estimation fusing video and inertial sensors. Proceedings of the British Machine Vision Conference 2017.

[32] Gall J , Rosenhahn B , Brox T , Seidel H-P (2006) Learning for multi-view 3D tracking in the context of particle filters. Advances in Visual Computing pp.59–69.

[33] Mathis A , Schneider S , Lauer J , Mathis MW (2020) A primer on motion capture with deep learning: principles, pitfalls, and perspectives. Neuron 108, 44–65. 33058765 10.1016/j.neuron.2020.09.017

[34] Wang J , Tan S , Zhen X , Xu S , Zheng F , He Z , Shao L (2021) Deep 3D human pose estimation: A review. Comput Vis Image Underst 210, 103225.

[35] Guler RA , Neverova N , Kokkinos I (2018) DensePose: dense human pose estimation in the wild. 2018 IEEE/CVF Conference on Computer Vision and Pattern Recognition.

[36] Kocabas M , Huang C-HP , Hilliges O , Black MJ (2021) PARE: Part Attention Regressor for 3D Human Body Estimation. InProceedings of the International Conference on Computer Vision (ICCV) pp11127–11137.

[37] Shapovalov R , Novotny D , Graham B , Labatut P , Vedaldi A (2021) DensePose 3D: Lifting canonical surface maps of articulated objects to the third dimension. In Proceedings of the IEEE/CVF International Conference on Computer Vision pp11729–11739.

[38] Blackwell S , Robinette KM , Boehmer M , Fleming S , Kelly S , Brill T , Hoeferlin D , Burnsides D , Daanen H (2002) , Air Force Research Laboratory, Human Effectiveness Directorate, Crew System Interface Division, Air Force Materiel Command, Civilian American and European Surface Anthropometry Resource (CAESAR): Final Report: Descriptions.

[39] SizeUSA dataset.

[40] Wolpert DH , Macready WG (1997) No free lunch theorems for optimization. IEEE Trans Evol Comput 1, 67–82.

[41] Sun C , Shrivastava A , Singh S , Gupta A (2017) Revisiting unreasonable effectiveness of data in deep learning era. 2017 IEEE International Conference on Computer Vision (ICCV).

[42] Wang C , Zhang F , Zhu X , Ge SS (2022) Low-resolution human pose estimation. Pattern Recognit 126, 108579.

[43] Xiao B , Wu H , Wei Y (2018) Simple baselines for human pose estimation and tracking. Proceedings of the European Conference on Computer Vision (ECCV).

[44] Batini C , Scannapieco M (2016) Data and Information Quality, Springer International Publishing.

[45] Ionescu C , Papava D , Olaru V , Sminchisescu C (2014) Human3.6M: Large scale datasets and predictive methods for 3D human sensing in natural environments. IEEE Trans Pattern Anal Mach Intell 36, 1325–1339. 26353306 10.1109/TPAMI.2013.248

[46] Sigal L , Balan AO , Black MJ (2010) HumanEva: Synchronized video and motion capture dataset and baseline algorithm for evaluation of articulated human motion. Int J Comput Vision 87, 4–27.

[47] Mehta D , Rhodin H , Casas D , Fua P , Sotnychenko O , Xu W , Theobalt C (2017) Monocular 3D human pose estimation in the wild using improved CNN supervision. In2017 International Conference on 3D Vision (3DV) pp506–516.

[48] Mahmood N , Ghorbani N , Troje NF . AMASS: Archive of motion capture as surface shapes. Proc Estonian Acad Sci Biol Ecol.

[49] Ionescu C , Li F , Sminchisescu C (2011) Latent structured models for human pose estimation. In 2011 International Conference on Computer Vision, pp. 2220–2227.

[50] von Marcard T , Henschel R , Black MJ , Rosenhahn B , Pons-Moll G (2018) Recovering accurate 3d human pose in the wild using imus and a moving camera. InProceedings of the European Conference on Computer Vision (ECCV), pp. 601–617.

[51] Ostrek M , Rhodin H , Fua P , Müller E , Spörri J (2019) Are existing monocular computer vision-based 3D motion capture approaches ready for deployment? A methodological study on the example of alpine skiing. Sensors (Basel) 19, 4323. 31590465 10.3390/s19194323 PMC6806076

[52] Leroy V , Weinzaepfel P , Bregier R , Combaluzier H , Rogez G (2020) SMPLy benchmarking 3D human pose estimation in the wild. 2020 International Conference on 3D Vision (3DV).

[53] Mathis A , Mamidanna P , Cury KM , Abe T , Murthy VN , Mathis MW , Bethge M (2018) DeepLabCut: markerless pose estimation of user-defined body parts with deep learning. Nat Neurosci 21, 1281–1289. 30127430 10.1038/s41593-018-0209-y

[54] He K , Gkioxari G , Dollár P , Girshick R (2017) Mask R-CNN. In 2017 IEEE International Conference on Computer Vision (ICCV), pp. 2980–2988.

[55] Lin T-Y , Dollar P , Girshick R , He K , Hariharan B , Belongie S (2017) Feature pyramid networks for object detection. 2017 IEEE Conference on Computer Vision and Pattern Recognition (CVPR).

[56] Cao Z , Simon T , Wei S-E , Sheikh Y (2017) Realtime multi-person 2D pose estimation using part affinity fields. 2017 IEEE Conference on Computer Vision and Pattern Recognition (CVPR). 10.1109/TPAMI.2019.2929257 31331883

[57] Iansek R , Danoudis M (2017) Freezing of gait in Parkinson’s disease: its pathophysiology and pragmatic approaches to management. Mov Disord Clin Pract 4, 290–297. 30868095 10.1002/mdc3.12463 PMC6407046

[58] Yang Y , Ramanan D (2013) Articulated human detection with flexible mixtures of parts. IEEE Trans Pattern Anal Mach Intell 35, 2878–2890. 24136428 10.1109/TPAMI.2012.261

[59] Andriluka M , Pishchulin L , Gehler P , Schiele B (2014) 2D human pose estimation: new benchmark and state of the art analysis. 2014 IEEE Conference on Computer Vision and Pattern Recognition.

[60] Sibley KG , Girges C , Hoque E , Foltynie T (2021) Video-based analyses of Parkinson’s disease severity: a brief review. J Parkinsons Dis 11, S83–S93. 33682727 10.3233/JPD-202402 PMC8385513

[61] Erb MK , Kelley Erb M , Karlin DR , Ho BK , Thomas KC , Parisi F , Vergara-Diaz GP , Daneault J-F , Wacnik PW , Zhang H , Kangarloo T , Demanuele C , Brooks CR , Detheridge CN , Kabiri NS , Bhangu JS , Bonato P (2020) mHealth and wearable technology should replace motor diaries to track motor fluctuations in Parkinson’s disease. NPJ Digit Med 3, 6. 31970291 10.1038/s41746-019-0214-x PMC6969057

[62] Galna B , Barry G , Jackson D , Mhiripiri D , Olivier P , Rochester L (2014) Accuracy of the Microsoft Kinect sensor for measuring movement in people with Parkinson’s disease. Gait Posture 39, 1062–1068. 24560691 10.1016/j.gaitpost.2014.01.008

[63] Siena FL , Byrom B , Watts P , Breedon P (2018) Utilising the Intel RealSense camera for measuring health outcomes in clinical research. J Med Syst 42, 53. 29404692 10.1007/s10916-018-0905-x PMC5799357

[64] Brooks C , Eden G , Chang A , Demanuele C , Kelley Erb M , Shaafi Kabiri N , Moss M , Bhangu J , Thomas K (2019) Quantification of discrete behavioral components of the MDS-UPDRS. J Clin Neurosci 61, 174–179. 30385169 10.1016/j.jocn.2018.10.043

[65] Aquino CC , Fox SH (2015) Clinical spectrum of levodopa-induced complications. Mov Disord 30, 80–89. 25488260 10.1002/mds.26125

[66] Lu M , Poston K , Pfefferbaum A , Sullivan EV , Fei-Fei L , Pohl KM , Niebles JC , Adeli E (2020) Vision-based estimation of MDS-UPDRS gait scores for assessing Parkinson’s disease motor severity. Med Image Comput Comput Assist Interv 12263, 637–647. 33103164 10.1007/978-3-030-59716-0_61 PMC7585545

[67] Lu M , Zhao Q , Poston KL , Sullivan EV , Pfefferbaum A , Shahid M , Katz M , Kouhsari LM , Schulman K , Milstein A , Niebles JC , Henderson VW , Fei-Fei L , Pohl KM , Adeli E (2021) Quantifying Parkinson’s disease motor severity under uncertainty using MDS-UPDRS videos. Med Image Anal 73, 102179. 34340101 10.1016/j.media.2021.102179 PMC8453121

[68] Yin Z , Geraedts VJ , Wang Z , Contarino MF , Dibeklioglu H , van Gemert J (2022) Assessment of Parkinson’s disease severity from videos using deep architectures. IEEE J Biomed Health Inform 26, 1164–1176. 34310333 10.1109/JBHI.2021.3099816

[69] Lu M , Poston K , Pfefferbaum A , Sullivan EV , Fei-Fei L , Pohl KM , Niebles JC , Adeli E (2020) Vision-based estimation of MDS-UPDRS gait scores for assessing Parkinson’s disease motor severity. Med Image Comput Comput Assist Interv 12263, 637–647. 33103164 10.1007/978-3-030-59716-0_61 PMC7585545

[70] Marcante A , Di Marco R , Gentile G , Pellicano C , Assogna F , Pontieri FE , Spalletta G , Macchiusi L , Gatsios D , Giannakis A , Chondrogiorgi M , Konitsiotis S , Fotiadis DI , Antonini A (2020) Foot pressure wearable sensors for freezing of gait detection in Parkinson’s disease. Sensors 21, 128. 33379174 10.3390/s21010128 PMC7794778

[71] O’Sullivan SB , Schmitz TJ , Fulk G (2019) Physical Rehabilitation, F.A. Davis.

[72] Rabin ML , Earnhardt MC , Patel A , Ganihong I , Kurlan R (2016) Postural, bone, and joint disorders in Parkinson’s disease. Mov Disord Clin Pract 3, 538–547. 30363567 10.1002/mdc3.12386 PMC6178721

[73] Zheng Y , Zhang Y-J , Li X , Liu B-D (2012) Action recognition in still images using a combination of human pose and context information. In2012 19th IEEE International Conference on Image Processing pp785–788.

[74] Guo G , Lai A (2014) A survey on still image based human action recognition. Pattern Recognit 47, 3343–3361.

[75] Feng Y , Choutas V , Bolkart T , Tzionas D , Black MJ (2021) Collaborative regression of expressive bodies using moderation. In2021 International Conference on 3D Vision (3DV) pp792–804.

[76] Pishchulin L , Andriluka M , Schiele B (2014) Fine-grained activity recognition with holistic and pose based features. In Pattern Recognition, Springer International Publishing, pp. 678–689.

[77] Lage I , Ross AS , Kim B , Gershman SJ , Doshi-Velez F (2018) Human-in-the-loop interpretability prior, Adv Neural Inf Process Syst 31. https://proceedings.neurips.cc/paper/2018/file/0a7d83f084ec258aefd128569dda03d7-Paper.pdf. PMC7899143 33623354

[78] Bruckert S , Finzel B , Schmid U (2020) The next generation of medical decision support: a roadmap toward transparent expert companions. Front Artif Intell 3, 507973. 33733193 10.3389/frai.2020.507973 PMC7861251

[79] Fukui H , Hirakawa T , Yamashita T , Fujiyoshi H (2019) Attention branch network: Learning of attention mechanism for visual explanation. In Proceedings of the IEEE/CVF conference on computer vision and pattern recognition, pp. 10705–10714.

[80] Bronstein JM , Tagliati M , Alterman RL , Lozano AM , Volkmann J , Stefani A , Horak FB , Okun MS , Foote KD , Krack P , Pahwa R , Henderson JM , Hariz MI , Bakay RA , Rezai A , Marks WJ Jr , Moro E , Vitek JL , Weaver FM , Gross RE , DeLong MR (2011) Deep brain stimulation for Parkinson disease: an expert consensus and review of key issues. Arch Neurol 68, 165. 20937936 10.1001/archneurol.2010.260 PMC4523130

[81] Varol G , Romero J , Martin X , Mahmood N , Black MJ , Laptev I , Schmid C (2017) Learning from synthetic humans. In Proceedings of the IEEE conference on computer vision and pattern recognition openaccess.thecvf.com, pp. 109–117.

[82] Rogez G , Schmid C (2018) Image-based synthesis for deep 3D human pose estimation. Int J Comput Vis 126, 993–1008.

[83] Varol G , Laptev I , Schmid C , Zisserman A (2021) Synthetic humans for action recognition from unseen viewpoints. Int J Comput Vis 129, 2264–2287.

[84] Bolaños LA , Xiao D , Ford NL , LeDue JM , Gupta P , Doebeli C , Hu H , Rhodin H , Th M (2021) 3D virtual mouse-body generates synthetic training data for behavioral analysis. Nat Methods 18, 378–381. 33820989 10.1038/s41592-021-01103-9 PMC8034498

[85] Ríos VL , Ramalingasetty ST , Özdil PG , Arreguit J , Ijspeert AJ , Ramdya P (2021) NeuroMechFly, a neuromechanical model of adult Drosophila melanogaster, bioRxiv. 2021.04.17.440214. 10.1038/s41592-022-01466-7 35545713

