# Supplementary material for: Towards a Visualizable, De-identified Synthetic Biomarker of Human Movement Disorders
Source: J Parkinsons Dis. 2022 Oct 14;12(7):2085–96. doi: 10.3233/JPD-223351 (PMC10473142; doi:10.3233/JPD-223351)
Supplement: Supplementary Material [file jpd-12-jpd223351-s001.pdf]

Supplementary Material

Towards a Synthetic Visualizable, De-identified Synthetic Biomarker of Human Movement Disorders

Glossary

The definitions of some relevant terminology are provided. For a more general glossary, we refer to this site Machine Learning Glossary (https://developers.google.com/machine-learning/glossary).

Deep neural network: refers to a subset of artificial neural networks which contains multiple hidden layers.

Ground truth dataset: refers to a dataset with the correct answer. The correct answer can refer to a label, a vector, an image, etc.

Human interpretability: refers to the ability to provide a human understandable rationale for the decision process of a machine learning algorithm. It requires the algorithm to provide not only an accurate prediction but also an underlying reason for this prediction which is understandable by laymen in terms of machine learning.

Machine learning: refers to the study of computer algorithms that can learn directly from input data. Currently, the most common model used in this area is the artificial neural network.

Mean per joint position error (MEJPE): refers to the error that represents the misprediction of the joint by the algorithm to its actual position. Appendix B describes MEJPE in detail.

Overfitting: refers to a state at which the model was able to conform with the training dataset, whereas it lost its ability to make correct predictions on unseen data. The opposing state of overfitting is underfitting. In this state, the model failed to conform with the training data set. It may lead to low accuracy when applying this model both on training data and unseen data.

Over-parameterization: refers to a situation where having many different input parameters (such as body mesh points) can create ambiguities and does not necessarily result in a more accurate output during human pose estimation.

Regularization: a designed penalty term to prevent the model from becoming more and more complex during training. The inappropriate complexity can result in overfitting or underfitting.

Training dataset: refers to the dataset used during training. Another related term is the test dataset. It refers to the dataset reserved for validation of the model performance.

Metrics for evaluation of human pose estimation algorithms

The most widely used evaluation metric for 3D pose estimation algorithms is mean per joint position error (MPJPE) [1,2]. It measures the Euclidean distance between predictions and ground truth positions in millimeters. The MPJPE is defined by:

$$MPJPE = \frac{1}{N}\sum_{i=1}^{N} |J_i - J_i^*|_2$$

where the J_i denotes the position of ith joint given by the algorithm, and the J_i^* denotes the ground truth position of ith joint. Another evaluation metric is a 3D extension of percentage of correct keypoints (PCK) [1–4]. A joint will be considered as “correctly detected” if the Euclidean distance between the prediction and ground truth is lower than a fixed threshold.

The percentage of correct keypoints (PCK) and the area under the curve (AUC) are widely used to evaluate the performance of 2D pose estimators [2,5]. PCK refers to the percentage of joints where the distance (units: Pixel) between prediction and ground truth is less than a threshold usually defined by reference to a body part. In our case, the term PCK@0.2 is used when the threshold is defined as 0.2 times the torso diameter. Consistent with previous work, the torso diameter is defined as the Euclidean distance between the left shoulder and the right hip [6–8]. The term AUC on PCK@0.2 refers to the area under the curve of PCK@X with X between 0 and 0.2 with a step 0.002; as the threshold approaches 0, stringency will be increased and accuracy will suffer. Therefore, accuracy for both PCK and AUC range from 0% to 100%, with AUC reported to be significantly smaller than PCK. Based on previous studies, the average of AUC on the Leeds Sports Pose (LSP) test dataset ranges from 40% to 70% while PCK@0.2 ranges from 70% to 90% [9–11].

Supplementary Video 1: Illustration of different pose estimation methods

REFERENCES

[1] Wandt B, Rudolph M, Zell P (2021) CanonPose: Self-supervised monocular 3D human pose estimation in the wild. *Computer Vision and Pattern Recognition (CVPR).*

[2] Ben Gamra M, Akhloufi MA (2021) A review of deep learning techniques for 2D and 3D human pose estimation. *Image Vis Comput* **114**, 104282.

[3] Andriluka M, Pishchulin L, Gehler P, Schiele B (2014) 2D human pose estimation: new benchmark and state of the art analysis. *2014 IEEE Conference on Computer Vision and Pattern Recognition*.

[4] Yang Y, Ramanan D (2013) Articulated human detection with flexible mixtures of parts. *IEEE Trans Pattern Anal Mach Intell* **35**, 2878–2890.

[5] Leroy V, Weinzaepfel P, Bregier R, Combaluzier H, Rogez G (2020) SMPLy benchmarking 3D human pose estimation in the wild. *2020 International Conference on 3D Vision (3DV).*

[6] Toshev A, Szegedy C (2014) Deeppose: Human pose estimation via deep neural networks. In *Proceedings of the IEEE conference on computer vision and pattern recognition*, pp. 1653–1660.

[7] Chu SW, Song Y, Zouo JJ, Cai W (2019) Human pose estimation using deep convolutional Densenet hourglass network with intermediate points voting. In *2019 IEEE International Conference on Image Processing (ICIP)*, pp. 594–598.

[8] Ludwig K, Harzig P, Lienhart R (2022) Detecting arbitrary intermediate keypoints for human pose estimation with vision transformers. In *Proceedings of the IEEE/CVF Winter Conference on Applications of Computer Vision*, pp. 663–671.

[9] Yu X, Zhou F, Chandraker M (2016) Deep deformation network for object landmark localization. In *Computer Vision – ECCV 2016*, Springer International Publishing, pp. 52–70.

[10] Rafi U, Leibe B, Gall J, Kostrikov I (2016) An efficient convolutional network for human pose estimation. In *BMVC 2016*, p. 2.

[11] Zhang F, Zhu X, Ye M (2019) Fast human pose estimation. In *Proceedings of the IEEE/CVF Conference on Computer Vision and Pattern Recognition*, pp. 3517–3526.
